# Multibacillary Leprosy Presenting as Chronic Progressive Polyneuropathy Initially Diagnosed as Probable Chronic Inflammatory Demyelinating Polyneuropathy (CIDP): A Diagnostic Pitfall

**DOI:** 10.7759/cureus.117657

**Published:** 2026-10-08

**Authors:** Jhanvi Goyal, Sanjeev Gupta, Aneet Mahendra, Aditi Dabhra, Aayush Goel

**Affiliations:** 1 Dermatology, Maharishi Markandeshwar Institute of Medical Sciences and Research, Maharishi Markandeshwar (Deemed to be University), Ambala, IND

**Keywords:** chronic inflammatory demyelinating polyneuropathy, claw hand, hansen's disease, pure neuritic leprosy, neurology

## Abstract

Hansen disease is a neurocutaneous disease that may present predominantly with neurological manifestations. Chronic inflammatory demyelinating polyneuropathy (CIDP) is another condition that can present similarly to early neuritic Hansen disease. We report the case of a 60-year-old man who was initially diagnosed and treated as probable CIDP before subsequently being diagnosed with multibacillary lepromatous leprosy. This case illustrates the diagnostic challenges posed by overlapping neurological manifestations of Hansen disease and CIDP.

## Introduction

Although the global burden of Hansen disease has declined considerably with the widespread use of multidrug therapy, new cases continue to emerge, particularly in endemic countries, such as India [1]. While Hansen disease is traditionally regarded as a dermatological disorder, it has a marked predilection for peripheral nerves, and neurological manifestations may precede the appearance of characteristic skin lesions by months or even years [1-6].

Patients presenting with isolated or predominant neurological manifestations without skin lesions should be followed closely, particularly when a definitive other neurological diagnosis cannot be established. Hansen disease should remain an important differential diagnosis during follow-up, as early neuritic leprosy may closely resemble chronic inflammatory demyelinating polyneuropathy (CIDP) and other acquired neuropathies, making diagnostic distinction challenging [2-4,7,8]. The following case exemplifies the diagnostic dilemma arising from the clinical overlap between CIDP and Hansen disease.

## Case presentation

A 60-year-old man, who had been treated as a case of probable CIDP for the preceding five years, presented to our institute for neurological and dermatological evaluation.

Five years earlier, he had developed gradually progressive bilateral lower limb pain, followed by distal weakness and sensory impairment. As his neurological symptoms progressively worsened, he started treatment at a tertiary care centre, where he underwent extensive neurological evaluation, including electrophysiological studies, neuroimaging, comprehensive serological and immunological investigations, PET scan, and sural nerve biopsy. CSF examination revealed elevated protein levels (82.4 mg/dL), with a CSF glucose level of 54.9 mg/dL. Autoimmune evaluation, including antinuclear antibody (ANA), was negative. Nerve conduction studies demonstrated severe sensorimotor polyneuropathy, while sural nerve biopsy revealed severe axonal loss with secondary demyelination (Table 1).

**Table 1:** Serial nerve conduction study findings during the clinical course CMAP: compound muscle action potential; NCS: nerve conduction studies; SNAPs: sensory nerve action potentials

Date	Motor NCS	Sensory NCS	F-wave	Impression
Approximately 5 years 4 months before presentation	Left tibial and peroneal nerves normal. Right tibial nerve showed reduced CMAP, conduction velocity and amplitude. Right peroneal nerve showed reduced CMAP.	Bilateral sural nerves not recordable.	NR	Right lower limb motor axonal–demyelinating neuropathy with bilateral sensory axonal neuropathy.
Approximately 5 years 3 months before presentation	Bilateral median and ulnar nerves normal. Bilateral tibial and peroneal nerves showed prolonged distal latencies with reduced conduction velocities; reduced CMAP in right tibial and right peroneal nerves.	Left sural nerve normal; right sural nerve not recordable.	Right peroneal F-wave not recordable.	Suggestive of asymmetrical lower limb radiculopathy.
Approximately 4 years 6 months before presentation	CMAPs grossly preserved.	SNAPs grossly preserved.	Right median F-wave latency 24.95 ms (normal).	Sensorimotor axonal polyneuropathy.

In the absence of an alternative identifiable cause of neuropathy, he was diagnosed with probable CIDP and treated with intravenous immunoglobulin (IVIg) and immunosuppressants such as oral corticosteroids and oral azathioprine. Despite prolonged immunomodulatory therapy, there was no significant clinical improvement, and his neurological deficits continued to progress, resulting in hypothenar wasting, intrinsic hand muscle weakness, impaired grip strength, and claw hand deformity. After approximately one year of treatment, his condition reached a plateau, with persistent neurological deficits but no further progression. He remained in this state for the next five years before seeking a second opinion at the Neurology Department of our institution, where he was advised to continue the same treatment. During the same visit, the patient independently sought dermatological consultation after noticing a few reddish elevated lesions over his abdomen.

On detailed cutaneous examination, multiple erythematous infiltrated plaques were identified over the upper limbs, trunk, and back (Figures 1-2).

**Figure 1:**
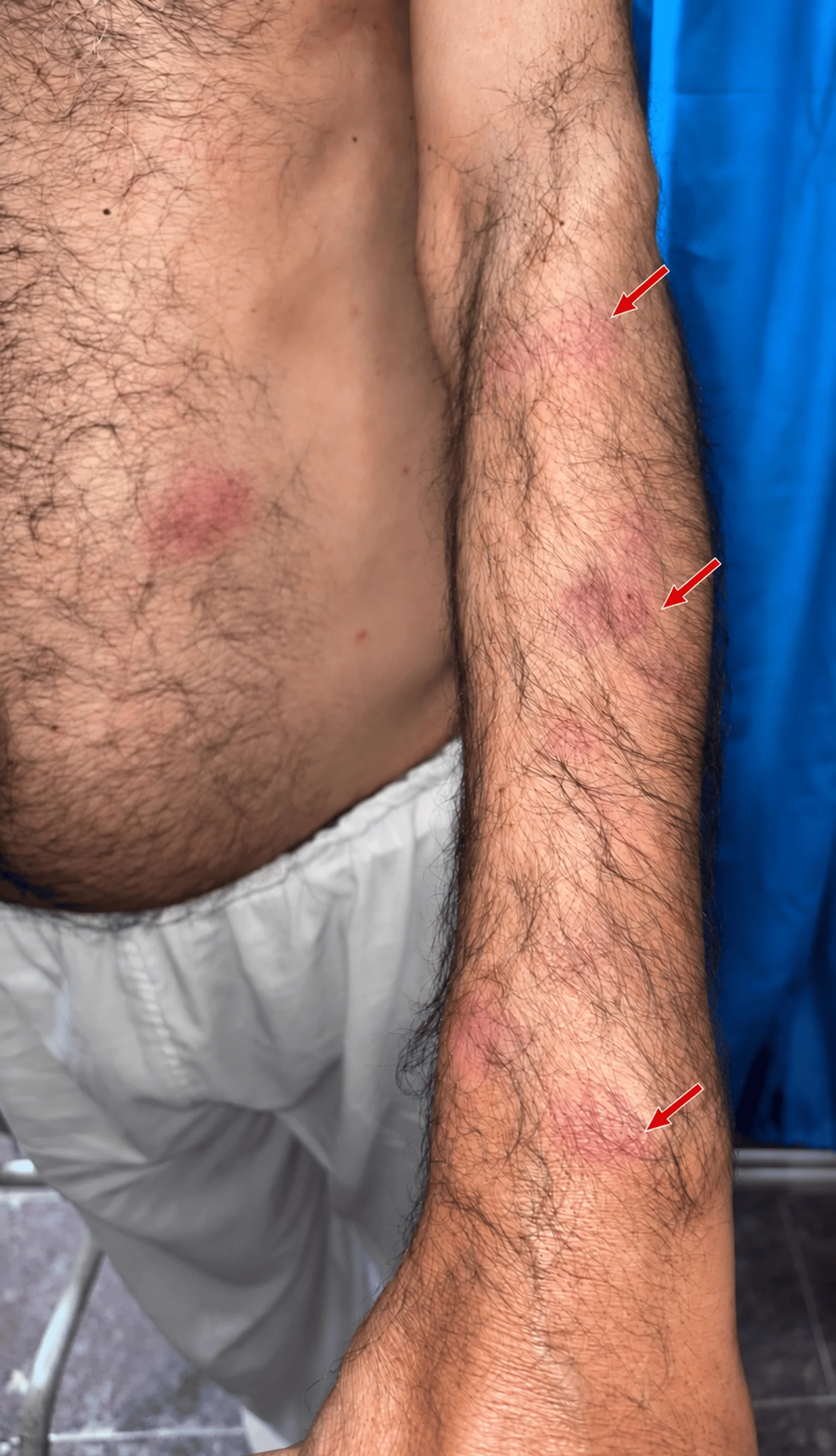
Erythematous infiltrated plaques over the abdomen and forearm, with ill-defined borders and infiltration

**Figure 2:**
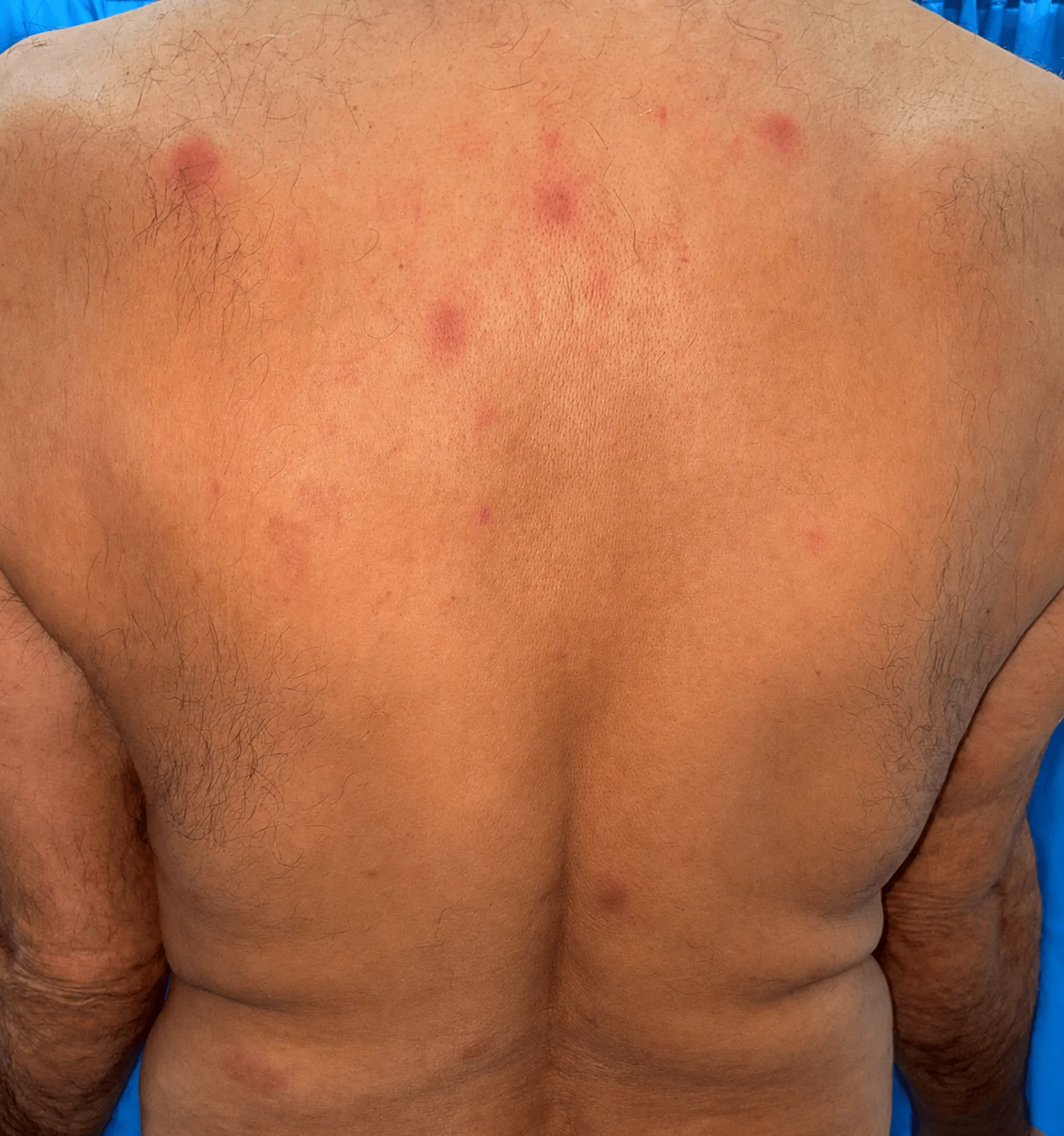
Multiple erythematous ill-defined infiltrated plaques over the back

As several of these lesions were located in areas not readily visible to the patient, he was unaware of their presence, and the exact time of onset could not be ascertained. On further examination, he also reported diminished touch and temperature sensation over the lesions. Peripheral nerve examination revealed bilaterally thickened ulnar, common peroneal, posterior tibial, and greater auricular nerves. The combination of chronic progressive neuropathy, sensory loss over the lesions, and multiple thickened peripheral nerves raised a strong clinical suspicion of Hansen disease.

Histopathological examination of a skin punch biopsy from a representative lesion demonstrated a thinned epidermis with a well-defined Grenz zone and diffuse dermal infiltrates of foamy histiocytes involving nerve twigs (Figure 3).

**Figure 3:**
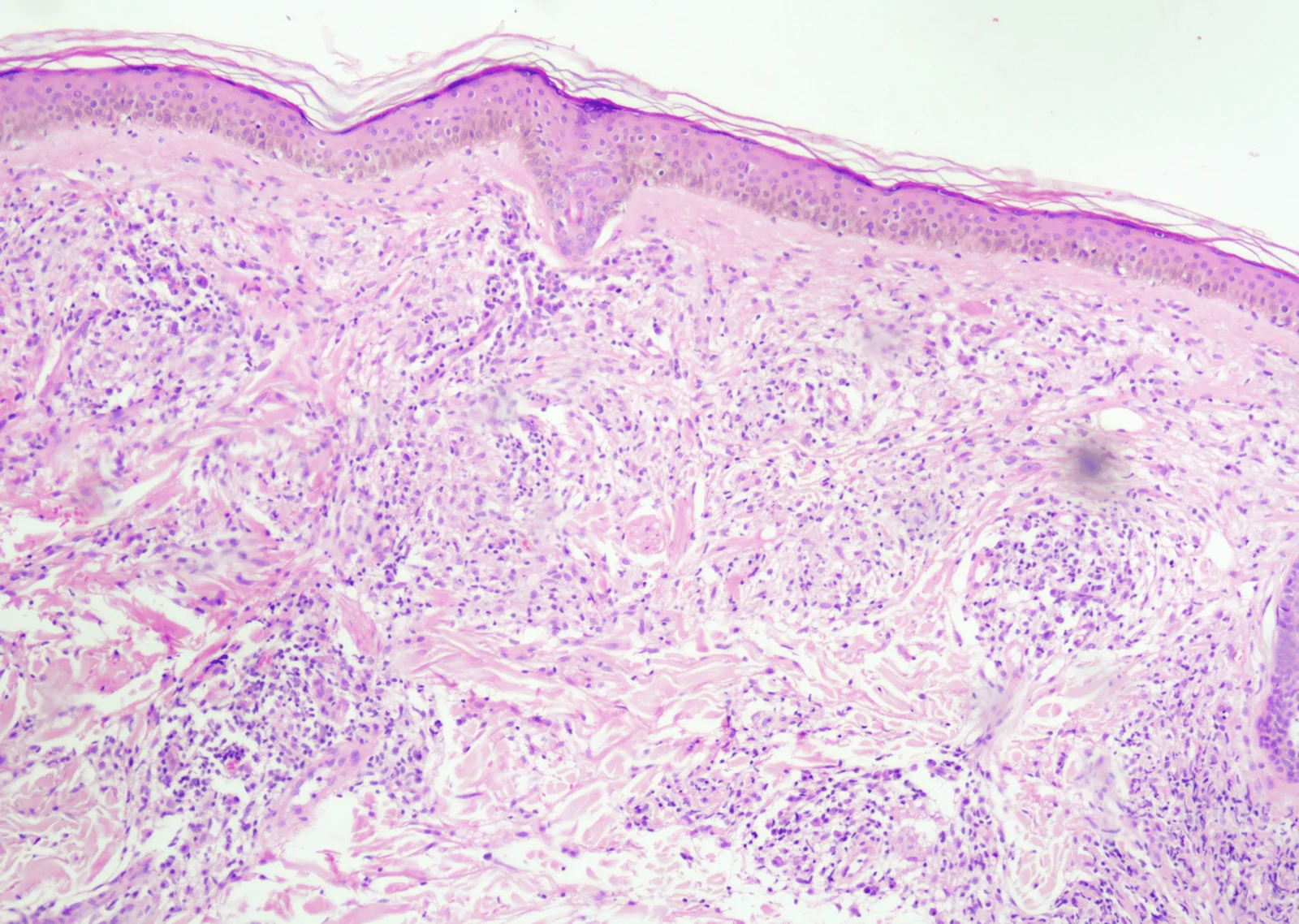
Hematoxylin and eosin (H&E) stain (low power ~10×) demonstrating sheets of foamy histiocytes admixed with lymphocytes within the dermis

Fite-Faraco staining demonstrated abundant acid-fast bacilli with a bacteriological index of 5+, supporting the diagnosis of multibacillary lepromatous leprosy (Figure 4).

**Figure 4:**
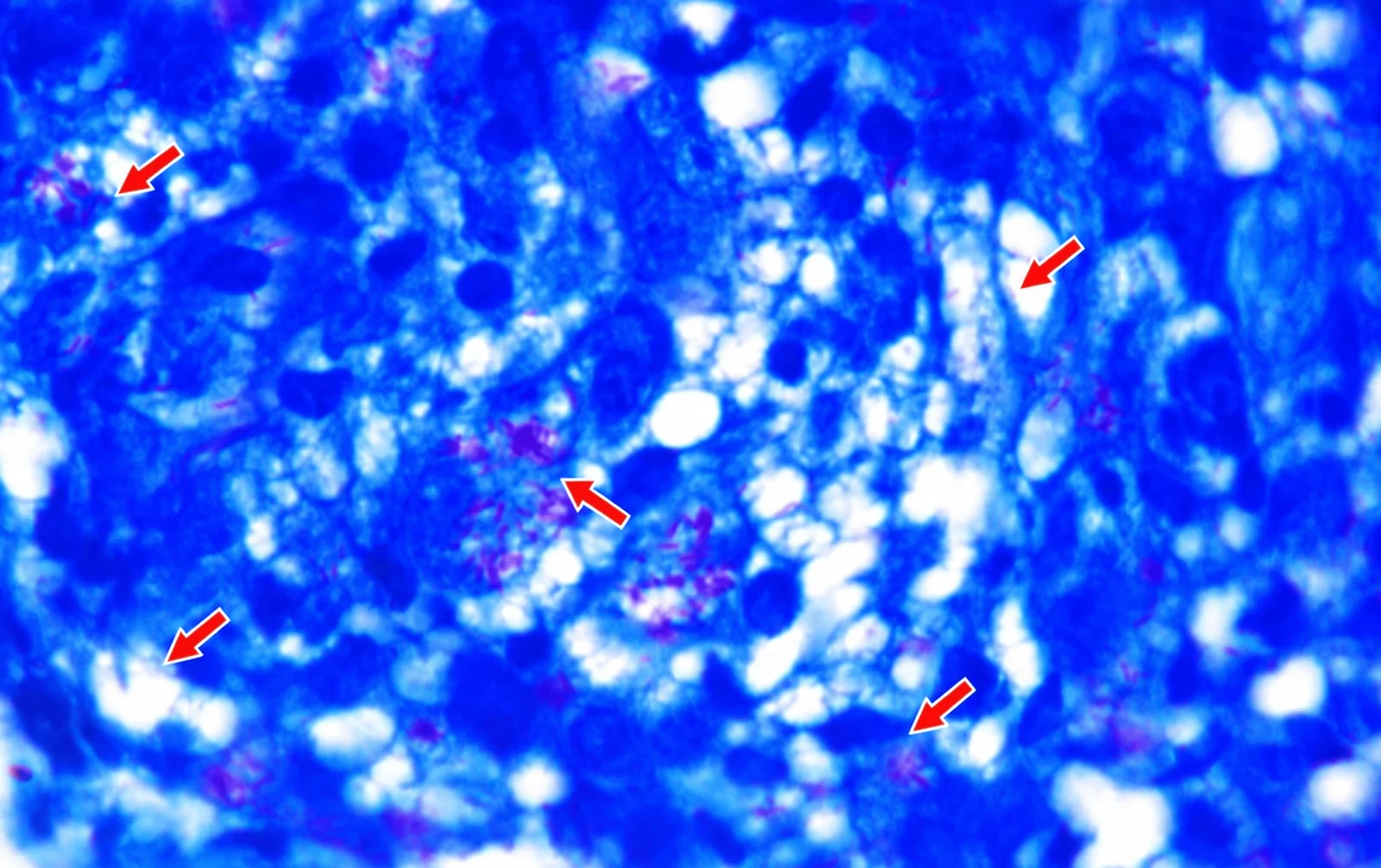
Fite-Faraco stain (×100, oil immersion) showing numerous acid-fast bacilli within foamy histiocytes, confirming multibacillary leprosy

Based on the clinical and histopathological findings, the disease was classified as lepromatous leprosy according to the Ridley-Jopling classification. Following confirmation of Hansen disease, the case was re-discussed with the Neurology Department to reassess the need for ongoing treatment for probable CIDP. A consensus was reached to discontinue the previously prescribed immunomodulatory therapy and commence multibacillary multidrug therapy (MB-MDT) alone. The patient is currently on the first MB-MDT pack along with other supportive management.

Limitations

The principal limitation of this report is the unavailability of the original neuroimaging, PET-CT, and sural nerve biopsy materials for retrospective review. Nevertheless, signed reports from the previously visited multiple tertiary care centres and other relevant medical records were available and were relied upon for documenting the patient's previous diagnostic workup.

## Discussion

Hansen disease's clinical presentation varies according to the host's cell-mediated immune response and encompasses a spectrum from borderline forms (BT, BB, and BL), representing intermediate disease, tuberculoid (TT) to lepromatous leprosy (LL), and pure neuretic leprosy. Nerve involvement is the hallmark of Hansen disease and the principal cause of long-term morbidity. *Mycobacterium leprae *exhibits a unique tropism for Schwann cells, resulting in progressive sensory and motor neuropathy. Neurological involvement may occur across the disease spectrum and present as mononeuropathy, mononeuritis multiplex, symmetrical polyneuropathy, or pure neuritic leprosy. Early recognition and treatment are essential to prevent irreversible nerve damage, deformity, and disability [2-5].

CIDP is another acquired immune-mediated neuropathy characterized by chronic progressive or relapsing symmetrical sensorimotor dysfunction with hyporeflexia or areflexia. Diagnosis relies on compatible clinical features supported by electrophysiological studies, cerebrospinal fluid analysis, nerve biopsy, and exclusion of alternative causes of neuropathy. Since no single investigation for CIDP is definitive, the diagnosis is established by integrating clinical, electrophysiological, and supportive laboratory findings and ruling out other neurological diseases [7,8]. Several cases of Hansen disease masquerading as CIDP have been reported in the literature, underscoring the diagnostic challenges posed by the overlapping neurological manifestations of these two conditions [8-10].

Our patient initially presented with isolated neurological manifestations and was diagnosed with probable CIDP. Hansen disease neuropathy may show electrophysiological features overlapping with CIDP, including slowing of nerve conduction and demyelinating changes; however, asymmetric nerve involvement and prominent axonal loss may favor Hansen disease. Misdiagnosis of Hansen disease as CIDP has been reported, although its frequency is not well established. Despite extensive investigations, including nerve biopsy, the diagnosis remained presumptive, emphasizing the limitations of relying solely on neurological investigations when the clinical picture is inconclusive. On retrospective review, the nerve conduction studies showed predominantly axonal and asymmetric abnormalities with limited demyelinating features, which were not strongly supportive of definite CIDP and could also be compatible with Hansen disease neuropathy. However, a complete dermatological examination was not performed at the initial presentation or during the subsequent five years of neurological follow-up; subtle cutaneous findings may therefore have been overlooked.

Whether Hansen disease was present from the outset, developed later, or coexisted with CIDP cannot be determined. The purpose of this report is not to suggest an association between the two conditions but to emphasize the importance of a thorough dermatological examination in patients with unexplained peripheral neuropathy when the neurological diagnosis remains uncertain. In our patient, an earlier cutaneous examination might have facilitated timely diagnosis. Consequently, it cannot be established retrospectively whether cutaneous manifestations were absent initially or remained unrecognized. This case therefore underscores an important clinical lesson: a complete dermatological examination is not a specialized investigation but a fundamental component of routine clinical assessment. In patients with unexplained or diagnostically uncertain peripheral neuropathy, careful examination of the skin should be performed at presentation and repeated during follow-up, particularly when the clinical course is atypical or the response to treatment is unsatisfactory.

## Conclusions

Hansen disease should remain an important differential diagnosis in patients with chronic progressive peripheral neuropathy in endemic regions, particularly when extensive evaluation fails to identify another cause or when neurological deficits continue to progress despite appropriate therapy. Early dermatological assessment, including careful examination of the skin and peripheral nerves, may facilitate timely diagnosis and prevent irreversible nerve damage and disability. In the present case, the available evidence does not definitively establish whether Hansen disease was present at the initial neurological presentation or subsequently developed or coexisted with CIDP.
